# Paracrine sonic hedgehog signalling by prostate cancer cells induces osteoblast differentiation

**DOI:** 10.1186/1476-4598-8-12

**Published:** 2009-03-02

**Authors:** Samantha M Zunich, Taneka Douglas, Maria Valdovinos, Tiffany Chang, Wade Bushman, David Walterhouse, Philip Iannaccone, Marilyn LG Lamm

**Affiliations:** 1 Department of Pediatrics, Northwestern University Feinberg School of Medicine, and Developmental Biology Program, Children's Memorial Research Center, 2300 Children's Plaza 204, Chicago, IL 60614, USA; 2 Department of Biological Sciences, Dartmouth College, Hanover, NH 03755, USA; 3 Division of Urology/Department of Surgery, University of Wisconsin, G5/350 Clinical Sciences Center, 600 Highland Avenue, Madison, WI 53705, USA

## Abstract

**Background:**

Sonic hedgehog (Shh) and components of its signalling pathway have been identified in human prostate carcinoma and increased levels of their expression appear to correlate with disease progression and metastasis. The mechanism through which Shh signalling could promote metastasis in bone, the most common site for prostate carcinoma metastasis, has not yet been investigated. The present study determined the effect of Shh signalling between prostate cancer cells and pre-osteoblasts on osteoblast differentiation, a requisite process for new bone formation that characterizes prostate carcinoma metastasis.

**Results:**

LNCaP human prostate cancer cells modified to overexpress Shh (designated LNShh cells) and MC3T3 mouse pre-osteoblasts were maintained as mixed populations within the same culture chamber. In this non-conventional mixed culture system, LNShh cells upregulated the expression of Shh target genes *Gli1 *and *Patched 1 *(*Ptc1*) in MC3T3 cells and this was inhibited by cyclopamine, a specific chemical inhibitor of hedgehog signalling. Concomitantly, MC3T3 cells exhibited time-dependent decreased cell proliferation, upregulated alkaline phosphatase *Akp2 *gene expression, and increased alkaline phosphatase activity indicative of early phase osteoblast differentiation. LNShh cell-induced differentiation was inhibited in MC3T3 cells stably transfected with a dominant negative form of Gli1, a transcription factor that mediates Shh signalling. Interestingly, LNShh cells did not significantly increase the endogenous expression of the osteoblast differentiation transcription factor *Runx2 *and its target genes *osteocalcin *and *osteopontin*. Consistent with these results, exogenous Shh peptide did not upregulate *Runx2 *expression in MC3T3 cells. However, *Runx2 *levels were increased in MC3T3 cells by ascorbic acid, a known stimulator of osteoblast differentiation.

**Conclusion:**

Altogether, these data demonstrate that Shh-expressing prostate cancer cells can directly and specifically induce differentiation in pre-osteoblasts via a Gli1-dependent mechanism that does not require transcriptional upregulation of *Runx2*. Paracrine activation of the Shh pathway in osteoblast progenitors and subsequent induction of osteoblast differentiation could be a mechanism through which high levels of Shh expression in prostate carcinoma contribute to bone metastasis. Targeting of paracrine Shh signalling may provide an effective therapeutic strategy against prostate carcinoma metastasis in bone.

## Background

Bone is the most common site for metastasis of prostate carcinoma affecting as many as 90% of patients with metastatic disease [1]. Complications from skeletal metastasis result in a dramatic reduction in the patient's quality of life and invariably lead to death [2,3]. A clear understanding of the factors and mechanisms that promote the migration of prostate cancer cells to bone, their colonization of the bone environment and subsequent formation of metastases is critically needed to prevent and effectively treat this disease. The commonly held "seed and soil" theory emphasizes the importance of the bone microenvironment in determining the colonization success of neoplastic "seed" cells [4]. On the other hand, prostate cancer cells can alter gene expression and cell functions in osteoblasts, suggesting that invading tumor cells might also influence the host bone milieu making it a favourable "soil" for metastasis [5,6]. This latter view fuels the on-going search for factors secreted by prostate cancer cells that might uniquely signal to bone and promote metastases.

Sonic hedgehog (Shh) is a secreted glycopeptide that plays essential functions in development and disease [7,8]. The binding of Shh to its transmembrane receptor Patched 1 (Ptc1) triggers the release of the transmembrane protein Smoothened (Smo) from Ptc1-mediated inhibition. Subsequent activation of the intracellular signalling cascade leads to the transcriptional regulation of Shh target genes by the Gli family of transcription factors: Gli1, Gli2, and Gli3.

Shh and members of its signalling pathway are expressed in prostate carcinoma and increased levels of their expression appear to correlate with cancer progression and metastasis [9-12]. Shh expression has been localized in prostatic epithelia or glandular lumens of prostate tumors indicating that epithelial-derived prostate cancer cells are a primary if not sole source of the Shh protein [9,11].

We have shown previously that Shh-expressing human prostate cancer cells upregulated *Gli1 *expression in surrounding tumor stroma and accelerated tumor growth in a mouse xenograft model of human prostate cancer [9]. This apparent paracrine activation of the pathway in prostate tumorigenesis is highly reminiscent of Shh-Gli signalling at epithelial-mesenchymal boundaries in several developing organ systems including the prostate that promotes cell proliferation and morphogenesis [13,14].

The mechanism by which Shh-Gli signalling could promote prostate carcinoma metastasis in bone has not been previously determined. The present study provides evidence of direct and specific paracrine Shh-Gli signalling between prostate cancer cells and pre-osteoblasts which leads to osteoblast differentiation, a requisite process for osteoblastic metastasis.

## Results

### Shh-expressing human prostate cancer cells, LNShh, directly activate hedgehog signalling in MC3T3 pre-osteoblasts

Paracrine interactions between cells have traditionally been studied in vitro by co-culturing cells in separate chambers while sharing the same culture medium. We have developed a non-conventional mixed culture system where different cell populations are cultured within the same chamber, thus, allowing them to establish physical associations that most likely occur in vivo and influence cell-cell signalling.

**Figure 1:**
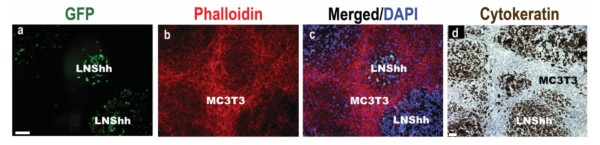
**Morphologic pattern of LNShh human prostate cancer cells and MC3T3 mouse pre-osteoblasts in mixed culture**. MC3T3 cells were mixed with Shh-expressing LNShh cells then seeded onto chamber slides and maintained for 14 days. (Panels a, b and c) GFP-expressing LNShh cells (stably transfected with pIRES2-h*Shh*-EGFP vector) formed clusters surrounded by a stroma of MC3T3 cells which expressed intense phalloidin staining. DAPI was used as counterstain. Cells were visualized by fluorescent microscopy. (Panel d) LNShh cells in mixed culture were further identified by positive immunocytochemical staining for human cytokeratin 8 which was not detected in surrounding MC3T3 cells. Hematoxylin was used as counterstain. Scale bars: 50 μm.

Shh-expressing LNCaP human prostate cancer cells obtained via stable transfection with h*Shh *cDNA cloned into a pIRES2-EGFP vector, designated as LNShh cells, or vector-transfected control LNCaP cells were cultured within the same culture chamber with MC3T3 mouse pre-osteoblasts. The cells in this mixed culture system established, in a time-dependent manner, a distinctive morphologic pattern. There were no apparent differences in the spatial organization of the mixed cultures of MC3T3 cells and either control LNCaP or LNShh cells, and only images of mixed cultures of MC3T3 and LNShh cells are shown in Figure 1. Beginning about two weeks of mixed culture, clusters of LNShh cells, identified by their GFP fluorescence, were surrounded by a compact "stroma" of MC3T3 cells which exhibited more intense phalloidin staining of their F-actin filaments (Figure 1, panels a, b and c). Identification of the epithelial-derived LNShh cells in mixed cultures was further confirmed by their positive immunostaining for human cytokeratin 8 (Figure 1, panel d). When cultured alone, LNShh cells also formed colonies which became interconnected over time forming a meshwork instead of remaining discrete as shown in mixed cultures. On the other hand, single cultures of MC3T3 cells which reached confluence at about two weeks formed a sheet of cells instead of the reticulate network evident in mixed cultures (data not shown).

To demonstrate activation of Shh signalling in pre-osteoblasts by Shh-expressing prostate cancer cells in mixed cultures, the expression of known Shh target genes *Gli1 *and *Ptc1 *were determined by quantitative real time RT-PCR analysis using species specific primer sequences (Table 1) which amplified these genes in mouse MC3T3 cells but not in the human prostate cancer cells. Thus, amplification of *Gli1 *and *Ptc1 *in mixed cultures is highly, if not solely, attributable to their expression in MC3T3 mouse pre-osteoblasts. Conversely, using primers that amplified genes in human but not mouse cells, we determined that expression of human *GLI1 *did not differ significantly between control LNCaP and LNShh cells cultured with MC3T3 cells suggesting the absence of significant autocrine or feedback paracrine regulation of the hedgehog pathway, at least under conditions used in these studies (data not shown). We have previously used species-specific primers in RT-PCR analysis to demonstrate the upregulation of mouse *Gli1 *in mouse xenografts of human prostate carcinoma [9].

**Table 1:** Sequences (5'- 3') of primers and probes used in quantitative real time RT-PCR analysis of mouse (m) and human (h) gene expression.

Gene	Forward Primer	Probe	Reverse Primer
m*Gli1*	GGAAGTCCTATTCACGCCTTGA	TCAAGACGCACCTTCGGTCGCAC	CAACCTTCTTGCTCACACATGTAAG

m*Ptc1*	CTCCAAGTGTCGTCCGGTTT	CGTGCCTCCTGGTCACACGAACAA	ACACCGTGGTCTGAGAGCTGTAC

m*Akp2*	TCAGGGCAATGAGGTCACATC	CGCTGGGCCAAGGATGCTGG	CACCCGAGTGGTAGTCACAATG

m*Runx2*	CGAAATGCCTCCGCTGTTAT	TAGCCAGGTTCAACGAT	CGCTCCGGCCCACAA

m*Ocn*	GGCCCTGAGTCTGACAAAGC	TTCATGTCCAAGCAGGAGGGCA	GCCGGAGTCTGTTCACTACCTT

m*Opn*	CATGAAGAGCGGTGAGTCTAAGG	TCCCTCGATGTCATCCCTGTTGCC	CTTTCCGTTGTTGTCCTGATCA

mGapdh	AACCTGCCAAGTATGATGACATCA	TGAAGCAGGCATCTGAGGGCCC	CTGTTGAAGTCGCAGGAGACAA

h*SHH*	AAGGACAAGTTGAACGCTTTGG	ATCTCGGTGATGAACCAGTGGCCAG	TCGGTCACCCGCAGTTTC

h*GLI1*	GGGCACCATCCATTTCTACAGT	AGCCCAAGAGGGAGCGGGAAGG	TCAGTCTGCTTTCCTCCCTGAT

h*GAPDH*	CGACAGTCAGCC GCATCTT	CGCCAGCCGAGCCACATCG	TGACCAGGCGCCCAATAC

**Figure 2:**
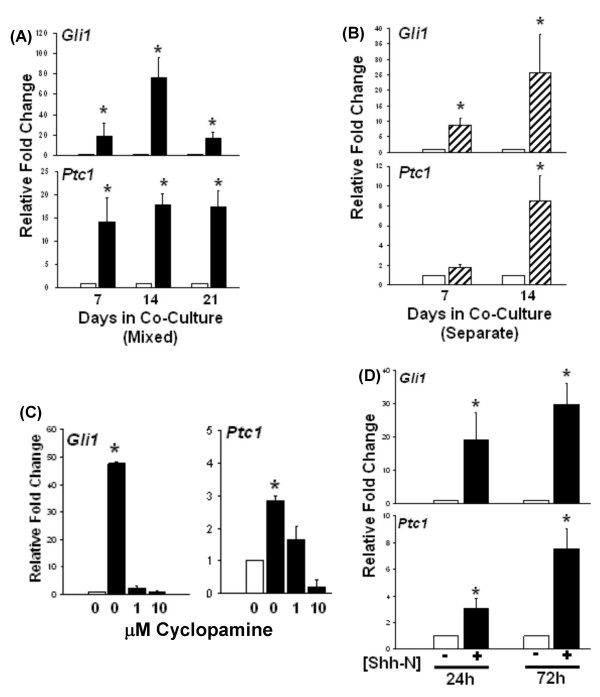
**LNShh cells upregulate *Gli1 *and *Ptc1 *expression in co-cultured MC3T3 cells**. In this and subsequent graphs, gene expression in MC3T3 cells was determined by quantitative real time RT-PCR analysis using mouse species-specific primers shown in Table 1. (A) MC3T3 cells were mixed with either LNCaP or LNShh cells then seeded onto 6-well plates (mixed cultures). Expression of *Gli1 *and *Ptc1 *in MC3T3 cells cultured with LNShh cells (filled bars) relative to those cultured with control LNCaP cells (open bars) were compared. (B) MC3T3 cells were grown on 6-well plates in the presence of culture inserts containing either LNCaP or LNShh cells (separate co-cultures). Expression of *Gli1 *and *Ptc1 *in MC3T3 cells co-cultured with LNShh cells (hatched bars) relative to those co-cultured with control LNCaP cells (open bars) were compared. (C) Effect of cyclopamine inhibition of paracrine Shh signalling. MC3T3 cells were maintained for 7 days in mixed culture with either LNCaP or LNShh cells in serum-free culture media without or with 1 μM and 10 μM cyclopamine. Expression of *Gli1 *and *Ptc1 *in MC3T3 cells cultured with LNShh cells in the absence or presence of cyclopamine (filled bars) relative to those cultured with control LNCaP cells without cyclopamine (open bars) were compared. (D) Effect of exogenous Shh peptide. MC3T3 cells were cultured alone in serum-free culture media without or with 1 μg/ml Shh-N (modified active N-terminal peptide of human Shh; kindly provided by Curis, Inc.). Relative expression of *Gli1 *and *Ptc1 *in MC3T3 cells treated without (open bars) or with (filled bars) Shh-N were compared. Data are means ± SD of 2–4 assays. *, *P *< 0.05.

MC3T3 pre-osteoblasts in mixed cultures with LNShh cells expressed increased levels of *Gli1 *and *Ptc1 *compared to those cultured with control LNCaP cells (Figure 2A). In a traditional co-culture system where cells were grown in separate chambers but shared the same culture medium, MC3T3 cells co-cultured with LNShh cells also expressed higher levels of *Gli1 *and *Ptc1 *(Figure 2B). Serum-free conditioned media from single confluent cultures of LNShh cells also increased *Gli1 *and *Ptc1 *expression in MC3T3 cells cultured alone which is consistent with the soluble nature of the secreted Shh ligand (data not shown).

Cyclopamine, a specific chemical inhibitor of hedgehog signalling through functional inhibition of the Shh receptor complex protein Smo, dramatically abrogated the increase in both *Gli1 *and *Ptc1 *expression in MC3T3 cells in mixed culture with LNShh cells (Figure 2C). Activation of the hedgehog pathway in MC3T3 cells was a direct and immediate effect of Shh action since exogenous Shh peptide (Shh-N) increased *Gli1 *and *Ptc1 *message as early as 24 h following treatment (Figure 2D).

These data demonstrate specific and direct paracrine activation of Shh signalling in pre-osteoblasts by Shh-expressing prostate cancer cells.

### LNShh cells induce early phase osteoblast differentiation

**Figure 3:**
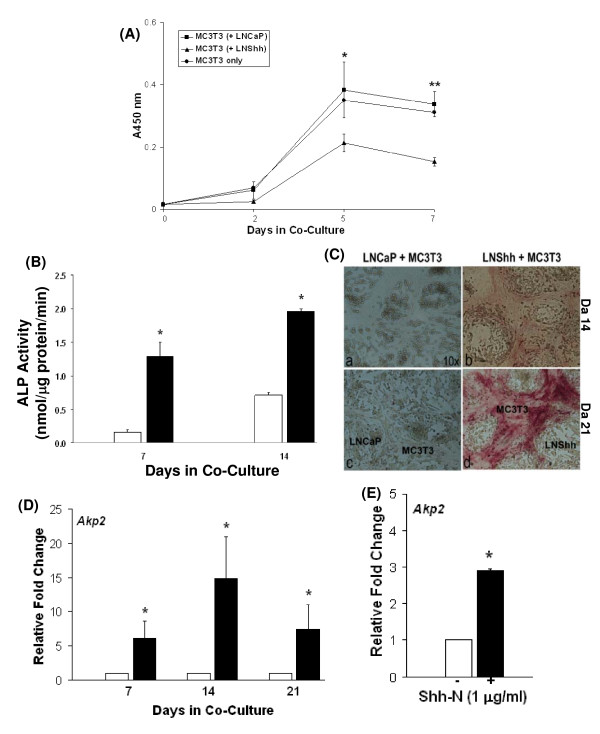
**LNShh cells induce osteoblast differentiation**. (A) Effect on cell proliferation. MC3T3 cells were grown in 24-well plates with culture inserts containing either control LNCaP cells (squares), LNShh cells (triangles), or no cells (circles). Absorbance measurements are in direct proportion to the number of living cells. Each point is the mean ± SD of 3 replicate wells, and results are representative of two independent experiments. *P < 0.05 and **P < 0.001, compared to MC3T3 cells co-cultured with LNShh cells at indicated time points. (B) Effect on ALP activity. Mixed cultures of MC3T3 cells with either control LNCaP or LNShh cells were grown in 6-well plates. Quantitative ALP activity was determined in cell lysates at indicated time points. ALP activity in mixed cultures of MC3T3 and LNShh cells (filled bars) were compared to those in mixed cultures of MC3T3 and control LNCaP cells (open bars). Data are means ± SD of 6 replicate determinations from 3 independent samples for each group. *, *P *< 0.05. (C) Localization of ALP activity. Mixed cultures of MC3T3 cells with either control LNCaP or LNShh cells were grown in 6-well plates and stained for ALP activity at indicated time points. Magnification, 10×. (D) Effect on *Akp2 *expression. MC3T3 cells were grown in mixed culture with either LNCaP or LNShh cells. Expression of *Akp2 *in MC3T3 cells cultured with LNShh cells (filled bars) relative to those cultured with control LNCaP cells (open bars) were compared. (E) MC3T3 cells were cultured alone in the absence (open bar) or presence (filled bar) of 1 μg/ml Shh-N for 24 h, and their relative *Akp2 *expression was compared. In (D) and (E), values are means ± SD of 2–5 assays. *, *P *< 0.05.

The ability of LNShh cells to induce osteoblast differentiation was examined. The initial phase of osteoblast differentiation is characterized by cell proliferation followed by growth arrest [15]. As shown in Figure 3A, proliferation of MC3T3 cells co-cultured with LNShh cells was significantly decreased by days 5 and 7 of co-culture compared to that of MC3T3 cells co-cultured with control LNCaP cells or cultured alone. These data suggest that Shh-expressing prostate cancer cells can inhibit cell proliferation in pre-osteoblasts.

Cell proliferation arrest is associated with a time-dependent increase in alkaline phosphatase (ALP) activity, a well-established indicator of osteoblast differentiation [15]. ALP activity in mixed cultures of MC3T3 and LNShh cells increased with time and was significantly higher relative to that in mixed cultures of MC3T3 and control LNCaP cells (Figure 3B). Staining for ALP activity was performed to determine the spatial localization of activity in mixed cultures. Consistent with data shown in Figure 3B, staining for ALP activity was greater in mixed cultures of LNShh and MC3T3 cells and intensified progressively through time (Figure 3C). Staining for ALP activity was localized almost exclusively in MC3T3 cells which surrounded clusters of LNShh cells (Figure 3C). Single cultures of MC3T3 cells also exhibited ALP staining when grown to confluence (data not shown).

Correspondingly, expression of the alkaline phosphatase gene *Akp2*, an early osteoblast differentiation marker gene, was increased in MC3T3 cells in mixed culture with LNShh cells, and this effect was mimicked by direct treatment of MC3T3 cells with exogenous Shh-N (Figures 3D and 3E, respectively).

MC3T3 cells in mixed culture with either control LNCaP or LNShh cells in ascorbic acid (AA)-free α-MEM media for 21 days were negative for both Alizarin red and Von Kossa staining indicating the absence of significant calcium deposition in and mineralization of the extracellular matrix (ECM), respectively (data not shown). These results are consistent with the known requirement for AA and inorganic phosphates for ECM mineralization during late stage osteoblast differentiation.

### Gli1 transcriptional activity is required for LNShh cell-induced osteoblast differentiation

To establish that LNShh cell-induced osteoblast differentiation occurred via the Shh signalling pathway, MC3T3 cells were stably transfected with human *GLI1 *cDNA which has a deletion in the region containing the trans activation domain: pCMV-*GLI(-)TAD *[16]. The translated GLI1 protein in transfected MC3T3 cells, designated as M-TAD cells, is expected to bind to the consensus DNA Gli binding site but not activate the pathway, thus, acting as a dominant negative form of the Shh signalling transcription factor.

**Figure 4:**
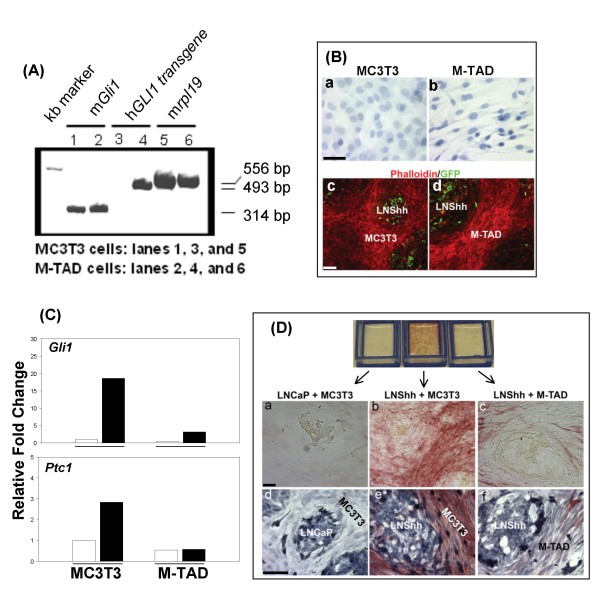
**Dominant negative Gli1 inhibits paracrine Shh signalling and osteoblast differentiation**. (A) MC3T3 cells were stably transfected with human *GLI1 *cDNA with deleted region containing the trans activation domain: pCMV-*GL1*(-)TAD. Expression of mouse endogenous *Gli1 *(m*Gli1*) and human *GLI1*-TAD transgene in parental MC3T3 cells and transfected cells, designated M-TAD cells, were determined by conventional RT-PCR analysis. The mouse gene for ribosomal protein 19 (m*rpl19*) was used as housekeeping gene. (B) Hematoxylin staining of single cultures of MC3T3 and M-TAD cells are shown in panels a and b, respectively. Fluorescent microscopy images of 14-day mixed cultures of MC3T3 and LNShh cells or M-TAD and LNShh cells are shown in panels c and d, respectively. The photomicrograph of mixed culture of MC3T3 and LNShh cells shown in panel c is the same as that shown in Figure 1 panel c, except only phalloidin staining and GFP expression are revealed here. Scale bars: 25 μm (a, b) and 50 μm (c, d). (C) MC3T3 cells or M-TAD cells were grown for 14 days in 6-well plates with culture inserts containing either LNCaP or LNShh cells (separate co-cultures). Expression of *Gli1 *and *Ptc1 *in MC3T3 or M-TAD cells co-cultured with LNShh cells (filled bars) relative to those co-cultured with control LNCaP cells (open bars) were compared. Results are representative of 2 independent experiments. (D) Mixed cultures of MC3T3 and M-TAD cells with either LNCaP or LNShh cells were grown in chamber slides for 14 days and stained for ALP activity (panels a, b and c) followed by hematoxylin staining (panels d, e and f). Scale bars: 50 μm.

Both parental MC3T3 and the M-TAD cells expressed endogenous mouse *Gli1 *mRNA (Figure 4A, lanes 1 and 2). However, only M-TAD cells expressed the message for the dominant negative human *GLI(-)TAD *transgene (Figure 4A, lanes 3 and 4). Surprisingly, the phenotype of M-TAD cells appeared different from that of parental MC3T3 cells. When grown to confluence, MC3T3 cells changed from a spindle-shaped phenotype to a cuboidal morphology with generally round nuclei indicative of the differentiated state (Figure 4B, panel a). M-TAD cells, on the other hand, retained their fusiform shape with nuclei elongated along the long cell axis consistent with an immature osteoblast state (Figure 4B, panel b). As with MC3T3 cells, M-TAD cells in mixed culture with LNShh cells formed a stroma surrounding human prostate cancer cells although their stroma appeared to be more compact (Figure 4B, panels c and d).

Expression of the dominant negative *GLI1 *effectively blocked the activation of the Shh pathway and inhibited the upregulation of *Gli1 *and *Ptc1 *in M-TAD cells cultured with LNShh cells (Figure 4C). Consequently, M-TAD cells cultured with LNShh cells for 14 days exhibited markedly decreased *Akp2 *expression (data not shown) and ALP activity (Figure 4D, panels a, b and c). Hematoxylin staining of mixed cultures following staining for ALP activity showed confluent populations of both MC3T3 and M-TAD cells in mixed cultures with LNShh cells indicating that the decrease in ALP activity in M-TAD cells was not a function of differences in cell confluence in the co-cultures (Figure 4D, panels d, e and f).

### LNShh cells do not upregulate the expression of *Runx2*

**Figure 5:**
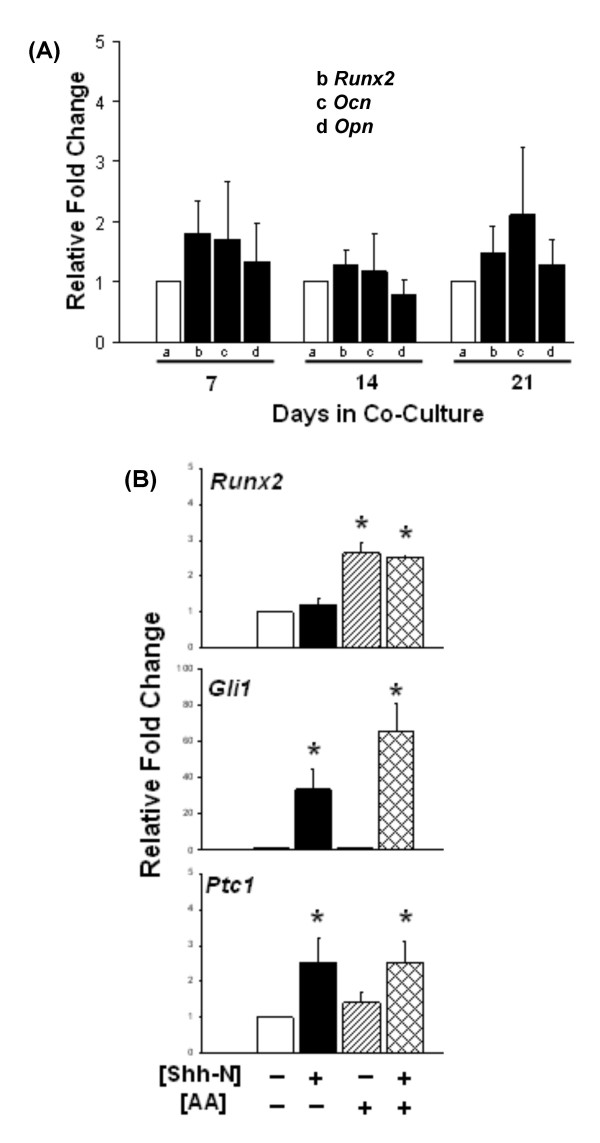
**LNShh cells do not upregulate *Runx2 *expression in MC3T3 cells**. (A) MC3T3 cells were grown in mixed cultures with either LNCaP or LNShh cells. Expression of *Runx2, Ocn*, and *Opn *in MC3T3 cells cultured with LNShh cells (filled bars) relative to those cultured with control LNCaP cells (open bars) were compared. (B) MC3T3 cells were cultured alone for 24 h without (-) or with (+) 1 μg/ml Shh-N or 50 μg/ml AA as indicated. Levels of expression of *Runx2*, *Gli1 *and *Ptc1 *among groups were compared. All values are means ± SD from 2–3 independent experiments. *, *P *< 0.05.

The transcription factor Runx2 plays a pivotal role in osteoblast differentiation and function [17,18]. The effect of LNShh cells on *Runx2 *expression in MC3T3 cells in mixed cultures was determined. Endogenous levels of *Runx2 *were not significantly changed in MC3T3 cells cultured with LNShh cells (Figure 5A, b relative to a). In addition, the expression of *Runx2 *target genes osteocalcin (*Ocn*) and osteopontin (*Opn*) were not significantly increased (Figure 5A, c and d, respectively, relative to a).

Consistent with these data, activation of the Shh pathway in MC3T3 cells by exogenous Shh-N, as evidenced by increased expression in *Gli1 *and *Ptc1*, did not upregulate *Runx2 *expression (Figure 5B). On the other hand, exogenous AA significantly increased *Runx2 *levels in MC3T3 cells without activating the Shh pathway (Figure 5B). Combined stimulation with Shh-N and AA did not significantly increase the expression of *Runx2 *relative to treatment with AA alone, or the expression of Shh target genes *Gli1 *and *Ptc1 *relative to treatment with Shh-N alone indicating the absence of synergistic interaction between these two factors. AA upregulates the expression of osteoblast differentiation marker genes including *Runx2 *and promotes osteoblast differentiation [19,20]. These data, therefore, indicate that early phase osteoblast differentiation in pre-osteoblasts induced by paracrine Shh signalling might proceed in a mechanism that does not require the transcriptional regulation of *Runx2*.

## Discussion

The functional relevance of upregulated Shh expression in human prostate carcinoma to the development of bone metastasis has not been previously investigated. We now provide data which indicate that prostate cancer cells that express high levels of Shh can directly and specifically activate the signalling pathway in osteoblast progenitors and induce osteoblast differentiation, an essential process in new bone formation that characterizes prostate carcinoma metastasis.

LNCaP human prostate cancer cells genetically engineered to overexpress Shh (designated LNShh cells) upregulated the expression of Shh-responsive target genes *Gli1 *and *Ptc1 *in co-cultured MC3T3 mouse pre-osteoblasts and this action was inhibited by cyclopamine, a specific chemical inhibitor of hedgehog signalling, and mimicked by exogenous Shh peptide. LNShh cell-induced activation of the Shh signalling pathway in pre-osteoblasts led to the induction of early phase osteoblast differentiation characterized by abrogated cell proliferation and increased expression and activity of alkaline phosphatase. Conversely, failure to activate the Shh pathway by a dominant negative form of the transcription factor Gli1 in pre-osteoblasts inhibited LNShh cell-induced osteoblast differentiation. These data confirm previous findings which have demonstrated a role for the hedgehog proteins, Shh and Indian hedgehog (Ihh), in osteoblast differentiation [21-27].

*Ihh*, *Ptc1*, *Smo*, *Gli1*, *Gli2*, and *Gli3 *are expressed in developing bone, and hedgehog signalling plays a vital role in skeletal development [28-35]. The expression of *Ptc1*, *Smo*, and *Gli1 *in perichondrial cells adjacent to *Ihh*-expressing prehypertropic chondrocytes strongly suggests a paracrine pathway through which hedgehog signalling might induce differentiation of perichondrial cells into osteoblasts during endochondral bone formation [21,30,34,35].

Our data suggest that the apparent paracrine pathway of hedgehog signalling in bone development may be recapitulated in bone metastasis where Shh-expressing prostate cancer cells activate the pathway in osteoblast progenitors and induce osteoblast differentiation. Reciprocally, differentiated osteoblasts might provide factors that are favourable to tumor growth in bone. Many identified downstream target genes of Gli1 transcriptional activity are known to regulate cell proliferation and cell adhesion [36].

The downstream mechanism through which Shh signalling induces osteoblast differentiation is not fully understood. *Runx2 *(also known as *Cbfa1*: *core-binding factor 1*) encodes a major and critical transcription activator of osteoblast differentiation [17,37,38]. A recent study has demonstrated that Ihh promotes osteoblast differentiation in multipotent mesenchymal cells C3H10T1/2 by upregulating the expression and function of *Runx2 *through the transcriptional action of Gli2 [27]. Our data, however, show that LNShh cell-induced early phase differentiation in MC3T3 pre-osteoblasts was not accompanied by a significant increase in endogenous levels of *Runx2 *and its downstream target genes *Ocn *and *Opn*. Moreover, exogenous Shh-N did not directly upregulate *Runx2 *expression in MC3T3 cells; whereas, the osteoblast differentiation factor AA increased *Runx2 *levels in MC3T3 cells without activating the Shh pathway. These data suggest that in cells that are already committed to the osteoblast lineage and express endogenous levels of *Runx2*, as is the case with MC3T3 cells, the induction of osteoblast differentiation by Shh signalling occurs through a mechanism that does not require further transcriptional upregulation of *Runx2*. Similarly, Xiao and co-workers concluded that osteoblast differentiation induced by bone morphogenetic protein (BMP) in mature osteoblast precursors does not require increased expression of *Cbfa1 *(*Runx2*) but might involve posttranscriptional regulation of the Runx2 protein [39]. In multipotent mesenchymal progenitors, however, Shh signalling promotes osteoblast commitment and differentiation through the upregulation of *Runx2 *message and activity [27,40].

Shh may act in concert with other factors in regulating osteoblast differentiation. Prostate cancer cells secrete signalling ligands including growth factors, bone morphogenetic proteins, and parathyroid hormone-related protein which are known to regulate osteoblast functions and whose actions have been linked to hedgehog signalling [5,6,24,41,42]. More studies are needed to tease out the functional interplay between hedgehog signalling and other pathways in the development of prostate carcinoma metastasis in bone.

Prostate cancer cells that metastasize to bone or those that are directly injected in bone in animal models will be in close physical contact with bone stromal cells including bone cells and their osteoblast progenitors, fibroblasts, hematopoetic cells, and multipotent mesenchymal stem cells. In the presence of mixed cell populations within the bone environment, it is difficult to isolate and determine direct cell-cell interactions that might impact the development of prostate carcinoma metastasis. The non-conventional mixed culture system developed in these studies provides an excellent in vitro model to investigate early outcomes of paracrine signalling between defined cell populations and to evaluate how these interactions might be impacted by other signalling pathways and/or therapeutic interventions.

## Conclusion

This study presents evidence that Shh-expressing prostate cancer cells directly and specifically activate the Shh signalling pathway in pre-osteoblasts. Paracrine Shh signalling in pre-osteoblasts leads to osteoblast differentiation via a Gli1-dependent mechanism that does not require transcriptional upregulation of endogenous *Runx2 *expression. These data provide a mechanism through which increased levels of Shh in human prostate carcinoma might specifically promote bone metastasis.

## Methods

### Plasmid Transfections

LNCaP human prostate cancer cells (ATCC, Rockville, MD) have been previously stably transfected with a 1.44 kb human *Shh *cDNA cloned into a pIRES2-EGFP mammalian cell expression vector (designated as LNShh cells) or with pIRES2-EGFP vector alone [9]. LNShh and control LNCaP cells were maintained at 37C, 5% CO_2 _in complete culture medium consisting of RPMI-1640 supplemented with 10% fetal bovine serum (FBS), 100 U/ml penicillin and 100 μg/ml streptomycin (Gibco Invitrogen). Shh gene and protein expression were routinely determined by quantitative real time RT-PCR and western blot analysis, respectively, and GFP expression was monitored by fluorescence microscopy.

Cells from the mouse calvaria-derived non-transformed pre-osteoblast cell line MC3T3-E1 (subclone 4; ATCC, Rockville, MD: designated as MC3T3 cells), were transfected with pCMV-*GLI*(-)TAD: a human *GLI1 *cDNA lacking a trans activation domain and cloned into pcDNA3 plasmid (kindly provided by Drs. Joon Won Yoon and David Walterhouse, Children's Memorial Research Center, Northwestern University Feinberg School of Medicine, Chicago, IL). The pCMV-*GLI*(-)TAD was previously prepared by deleting a 1,409-bp AccI fragment from the 3' end of human *GLI1 *cDNA [16]. MC3T3 cells were transfected with 3 μg DNA using the Effectene reagent according to manufacturer's protocol (Qiagen), selected for stable transfection with G418 (Sigma), and cloned by limiting dilution (designated M-TAD cells). Parental MC3T3 and M-TAD cells were maintained at 37C, 5% CO_2 _in non-differentiation complete culture medium consisting of ascorbic acid (AA)-free α-MEM supplemented with 10% FBS, 100 U/ml penicillin and 100 μg/ml streptomycin (Gibco Invitrogen).

### Mixed Culture of Cells

Control LNCaP or LNShh cells (5 × 10^4^) and MC3T3 cells (0.5 × 10^4^) were mixed in AA-free α-MEM complete culture medium and seeded per well of 6-well tissue culture plates. Cultures were maintained for the length of time specified in the experiments with media changes every 2–3 days.

### Separate Co-culture of Cells

Control LNCaP or LNShh cells (5 × 10^4^) were seeded per cell culture insert (transparent PET membrane, 0.4 μm pore size: BD Biosciences) in RPMI-1640 complete culture medium. MC3T3 cells (1 × 10^4^) were seeded per well of 6-well tissue culture plates in AA-free α-MEM complete culture medium. After two days, culture inserts grown with prostate cancer cells were transferred to wells grown with MC3T3 cells, and co-cultures were maintained in AA-free α-MEM complete culture medium for the length of time specified in the experiments with media changes every 2–3 days.

### RNA Isolation and Real Time Quantitative RT-PCR

Total RNA was extracted using Trizol (Invitrogen), purified using the RNeasy Mini Kit (Qiagen) and subjected to DNase treatment with RQ1 RNase-free DNase (Promega) to remove contaminating genomic DNA. The TaqMan^® ^Gold PCR Core Reagent Kit along with MuLV Reverse Transcriptase and RNase Inhibitor (Applied Biosystems) were used for cDNA synthesis. PCR primers (Invitrogen) and FAM-QSY7 probes (MegaBases, Inc.) for genes of interest and the housekeeping gene glyceraldehyde-3-phosphate dehydrogenase (*Gapdh*), whose sequences are shown in Table 1, were designed using the Primer Express 3.0 software program. mRNA expression was measured in duplicate or triplicate per sample using 40 cycles of amplification in the 7500 Fast Real-Time PCR System (Applied Biosystems). Reactions were routinely performed without Reverse Transcriptase to demonstrate RNA dependence of the reaction products. Results were analyzed using the comparative C_t _method. C_t _is the threshold cycle at which the reporter fluorescence signal for each amplicon passes above a fixed baseline. The average C_t _of the gene of interest was normalized to that of the housekeeping gene *Gapdh *to obtain the ΔC_t _for that gene. ΔC_t _values between experimental and control samples were then compared (ΔΔC_t_) to reflect the relative fold change in gene expression.

### Cell Proliferation Assay

Cells were maintained as separate co-cultures as described above. MC3T3 cells were seeded onto 24-well tissue culture plates at 0.2 × 10^4 ^cells per well. Control LNCaP or LNShh cells were seeded at 2 × 10^4 ^cells per cell culture insert. Following overnight incubation, culture inserts were transferred to wells and co-cultures were maintained in AA-free α-MEM complete culture medium with media changes every 2–3 days. At specified times, cell inserts were removed and proliferation of MC3T3 cells grown on wells was determined using the Cell Counting Kit-8 (Dojindo Laboratories, Japan) which is based on the formation of a water-soluble formazan dye through the activity of dehydrogenases in living cells.

### Immunocytochemistry and Immunofluorescence

Cells were maintained as mixed cultures in Lab-TekII CC2-treated chamber slides (Nunc) for the length of time specified in the experiments with media changes every 2–3 days. Cells were then fixed in 10% neutral buffered formalin for 10 minutes and permeated with 0.1% Triton X-100. For fluorescence immunocytochemistry, cells were stained with Alexa Flour 635 Phalloidin (Invitrogen: 1:40 dilution) and incubated in the dark for 20 minutes. Cells were counter-stained with 300 nM DAPI (Invitrogen). Slides were mounted using ProLong Gold antifade reagent (Invitrogen). For cytokeratin expression, fixed cells were stained with mouse monoclonal anti-human cytokeratin 8 antibody (DAKO: 1:50 dilution), developed with diaminobenzidine, and counterstained with hematoxylin. Slides were viewed in a Leica DMR-HC Upright Microscope and images were captured with imaging software (Improvision Openlab). Fluorescent images were captured at the following excitation/emission wavelengths (nm): Phalloidin-560/645; DAPI-360/470; GFP-480/527.

### Alkaline Phosphatase Activity

Quantitative determination of ALP activity was done using the p-Nitrophenyl Phosphate (pNPP) Liquid Substrate System according to manufacturer's protocol (Sigma Aldrich). Briefly, cells in culture wells were treated with 0.2% Triton X-100, harvested with a cell scraper, and subjected to freeze-thaw cycles. Lysates were centrifuged and supernatants (10 μg protein) were incubated with 150 μl pNPP for 5 h at room temperature in the dark. Absorbance at 405 nm was measured using a microplate reader, and ALP activity was calculated according to manufacturer's instructions. Protein determination was done using the Bio-Rad *DC *Protein Microplate Assay according to manufacturer's protocol.

For ALP staining, mixed cultures were fixed with 10% neutral buffered formalin for 10 minutes and incubated with alkaline phosphatase substrate solution for at least 30 minutes at room temperature in the dark. The substrate solution was prepared by adding Naphthol AS-MX Phosphate Alkaline Solution to a diazonium salt solution (Fast Violet B Salt capsule dissolved in distilled water) just before use (Sigma-Aldrich).

### Effect of Shh peptide

MC3T3 cells were seeded onto 6-well culture plates at 1 × 10^5 ^cells per well in AA-free α-MEM complete culture medium. Following overnight incubation, cells were treated with 1 μg/ml Shh-N, a modified active N-terminal peptide of human Shh (kindly provided by Curis Inc., Cambridge, MA), in serum-free AA-free α-MEM culture media for 24 h or 72 h.

### Inhibition of Shh Signalling by Cyclopamine

Cultures of mixed cells (as described above) were maintained for 6 days in serum-free AA-free α-MEM culture medium with 1 or 10 μM cyclopamine (vehicle controls were 0.01 or 0.1% ethanol, respectively). Cyclopamine, a generous gift from Dr. W. Gaffield (Western Regional Research Center, USDA, Albany, CA), has been previously used to inhibit Shh signalling in developing mouse prostate [43].

### Effect of Ascorbic Acid

Cultures of mixed cells (as described above) were maintained for 7 days in AA-free α-MEM complete culture medium without or with 50 μg/ml L-ascorbic acid (AA; Sigma). To determine the direct effect of AA, MC3T3 cells were seeded alone onto 6-well culture plates at 1 × 10^5 ^cells per well in AA-free α-MEM complete culture medium. Following overnight incubation, cells were treated without or with 50 μg/ml AA for 24 h.

### Data Analysis

Data were analyzed by ANOVA and pairwise multiple comparisons were done using the Bonferroni t-test at *P *< 0.05. Data are presented as means ± SD of at least 2 assays from independent experiments, each assay done in duplicate or triplicate.

## Competing interests

The authors declare that they have no competing interests.

## Authors' contributions

SMZ performed experiments, contributed to gene expression data analysis, and helped with manuscript preparation. TD performed experiments, generated the M-TAD cells, and contributed to gene expression data analysis. TC and MV performed some experiments and contributed to data analysis, and TC additionally performed the fluorescent microscopy imaging. WB provided the LNShh cells, contributed to data analysis and editing of the manuscript. DW provided the pCMV-*GLI*(-) TAD plasmid, contributed to data analysis and editing of the manuscript. PI contributed to the analysis of data and editing of the manuscript. MLGL designed the study, performed some experiments, performed data analysis, and prepared the manuscript. All authors read and approved the final manuscript.
